# Perioperative outcomes in different anesthesia techniques for patients undergoing hip fracture surgery: a systematic review and meta-analysis

**DOI:** 10.1186/s12871-023-02150-9

**Published:** 2023-05-27

**Authors:** Bo Ma, Haibiao Xie, Huayong Ling, Wuhua Ma

**Affiliations:** 1 Department of Anesthesiology, The First Affiliated Hospital of Guangzhou University of Chinese Medicine, Guangzhou, 510405 People’s Republic of China; 2 Department of Urology, Guangdong Provincial People’s Hospital (Guangdong Academy of Medical Sciences), Southern Medical University, Guangzhou, 510080 People’s Republic of China

**Keywords:** General anesthesia, Regional anesthesia, Hip fractures, Mortality, Pneumonia, Delirium

## Abstract

**Background:**

Previous studies of the perioperative effects of general and regional anesthesia in adult patients undergoing effects of different anesthesia techniques on patients undergoing hip fracture surgery have not produced consistent results. The aim of this systematic review and meta-analysis was to compare the hip fracture surgery.

**Methods:**

We performed a systematic review and meta-analysis to compare the effects of general anesthesia with regional anesthesia on in-hospital mortality, 30-day mortality, postoperative pneumonia, and delirium in adult hip fracture patients (≥ 18 years). Between January 1, 2022, and March 31, 2023, a systematic search was performed for retrospective observational and prospective randomized controlled studies in PubMed, Ovid Medline, Cochrane Library, and Scopus.

**Results:**

Twenty-one studies including 363,470 patients showed higher in-hospital mortality in the general anesthesia group compared with regional anesthesia (OR = 1.21; 95% CI 1.13–1.29; *P* < 0.001, *n* = 191,511). The 30-day mortality (OR = 1.00; 95% CI 0.96–1.05; *P* = 0.95, *n* = 163,811), the incidence of postoperative pneumonia (OR = 0.93; 95% CI 0.82–1.06; *P* = 0.28, *n* = 36,743) and the occurrence of postoperative delirium in the two groups (OR = 0.94; 95% CI 0.74–1.20; *P* = 0.61, *n* = 2861) had no significant difference.

**Conclusion:**

Regional anesthesia is associated with reduced in-hospital mortality. However, the type of anesthesia did not affect the occurrence of 30-day mortality, postoperative pneumonia, and delirium. A large number of randomized studies are needed in the future to examine the relationship between type of anesthesia, postoperative complications, and mortality.

**Supplementary Information:**

The online version contains supplementary material available at 10.1186/s12871-023-02150-9.

## Background

Due to the aging population, the absolute total number of hip fractures among people aged 55 years and older increased approximately four-fold between 2012 and 2016 in China [1]. With the 1.6 million hip fracture surgeries performed each year worldwide from 2016 to 2020, the risk of death within 30 days after surgery increased by 6.7% to 8.2% [2]. What is more, hip fracture surgery is expected to increase to 6.25 million per year by 2050 [3].

Previous studies to date have not provided sufficient evidence to determine the ideal mode of anesthesia. Studies evaluating general anesthesia versus regional anesthesia have produced inconsistent results when mortality was included as the primary outcome. Previous systematic reviews aimed at assessing this issue have been limited by the inadequate samples and generally low quality of randomized trials. In the past two years, large-scale high-quality randomized controlled studies have emerged, focusing on evaluating the impact of regional anesthesia and general anesthesia on the incidence of delirium in patients after hip fracture surgery [4, 5]. To integrate the impact of different anesthesia ways on post-surgery complications in previous studies, now we performed a meta-analysis of this result.

This systematic review and meta-analysis aimed to identify studies in the context of the type of anesthesia in patients undergoing hip fracture surgery. In-hospital mortality, pneumonia, and delirium were reviewed in recent 20 years in order to assess the effect of different anesthesia techniques after hip fracture in prospective randomized and retrospective observational studies.

## Methods

The study protocol has not been published before. This systematic review and meta-analysis adhered to the preferred reporting item of the guidelines for systematic reviews and meta-analysis [6]. The systematic evaluation and meta-analysis of observational studies followed the MOOSE (Meta-analysis of Observational Studies in Epidemiology) guidelines. It has been registered in the international prospective register of systematic reviews (Prospero: CRD42022372145).

### Inclusion and exclusion criteria

All the authors identified exclusion and inclusion criteria in advance before conducting a systematic review and meta-analysis. This systematic review and meta-analysis focused on the latest research evaluating modern anesthesia techniques. We included only human studies published between January 1, 2002, and March 31, 2023, evaluating the perioperative outcomes of general anesthesia versus regional anesthesia in adults with hip fractures. Prospective and retrospective randomized trials and observational studies were eligible for this review which addressed the incidence of in-hospital mortality, 30-day mortality, postoperative pneumonia, and delirium. Articles were excluded if the outcome parameters did not fit the outcome variables of this study. Case series, Case reports, systematic reviews, and meta-analysis were excluded.

### Literature searches

A systematic search was performed through PubMed, Ovid Medline, Cochrane Library, and Scopus. In PubMed, the full search strategy was: (((anesthesia [Mesh]) OR (anesthesia)) OR (anaesthesia)) AND (((((hip fractures [Mesh]) OR (hip fractures)) OR ((hip) AND (fractures))) OR (hip fracture)) OR ((hip) AND (fracture))) AND ((2002/1/1[PDAT]: 2023/3/31[PDAT])). The Detail literature search strategies in different databases were displayed in the form of Supplemental 1.

Study selection was based on independent screening of titles and abstracts in initial searches by two researchers (BM and HX). Qualified studies were independently reviewed in full by the same two reviewers for eligibility. Disagreement on study eligibility was discussed and resolved by consultation with the third author (HL).

### Data extraction

Data extraction from the included literature was executed independently by two investigators (BM and HX). For each eligible study, the information about the first author, country, type of study, anesthesia technique, detailed method of local anesthesia (technology, mode of administration, drug type, concentration, and dose), and the diagnostic criteria of complications were collected and recorded in Table 1. The information related to in-hospital mortality, 30-day mortality, the rate of postoperative pneumonia and delirium, and the main conclusions was collected and recorded in Table 2. The meta-analysis included at least two randomized or non-randomized studies comparing general anesthesia and regional anesthesia. The primary outcomes were mortality including in-hospital mortality and 30-day mortality. The secondary outcomes were the rate of postoperative pneumonia and delirium. The results of the different groups were reported in the same way. The number of events was extracted for dichotomous outcomes and the mean and standard deviation were extracted for continuous outcomes.

**Table 1:** Demographic characteristics of the included studies

Study (1st author year)	Country	Study type	Sample Size and Groups	RA		The diagnostic criteria for complications
				Single or continuous, type	Drug type, concentration and dose	
Basques [7] 2015	USA	Retrospective observational studies	9842 GA 7253 RA 2589	SA	Not mentioned	By ACS-NSQIP database
Benjamin [8] 2021	France	Retrospective observational studies	129 GA 43 RA 86	MNB 43 CSA 43	MNB: ropivacaine, 0.33%, 0.2–0.25 ml/kg CSA: bupivacaine 0.5%, 1.5 ml	MI: be confirmed by the cardiologist in charge. Hypoxemia: defined by the need for oxygen
Brox [9] 2016	USA	Retrospective observational studies	7316 GA 4257 RA 3059	Single SA CEA/CSEA NB	Not mentioned	/
Chu [10] 2015	China	Retrospective observational studies	104,088 GA 52044 RA 52044	NA	Not mentioned	Using ICD-9-CM diagnosis
Elisabetta [11] 2014	USA	Retrospective observational studies	68,493 GA 61554 RA 6939	SA/EA	Not mentioned	/
Fields [12] 2015	USA	Retrospective observational studies	6628 GA 4813 RA 1815	SA	Not mentioned	According to ACS- NSQIP
Heidari [13] 2011	Iran	Randomized controlled studies	387 GA 197 RA 190	Single SA CEA	SA: Bupivacaine 0.5%, 3 ml CEA: Bupivacaine 0.5%, 25 ml	Be diagnosed by the consultant specialist
Helwani [14] 2015	USA	Retrospective observational studies	10,498 GA 5396 RA 5102	SA	Not mentioned	According to ACS- NSQIP
Li [4] 2022	China	Randomized controlled studies	942 GA 471 RA 471	Single SA EA CSA/CEA Single or continuousNB	Be at the discretion of the consultant anesthesiologist	According to ACS-NSQIP guidelines, Delirium: be based on Delirium Rating Scale-Revised-98
Linda [15] 2012	USA	Retrospective observational studies	308 GA 235 RA 73	Single SA CSA/CEA CSEA NB	Not mentioned	By reviewing the discharge summaries from the hospital’s electronic records
Neuman [16] 2012	USA	Retrospective observational studies	18,158 GA 12,904 RA 5,254	Not mentioned	Not mentioned	Using ICD-9-CM diagnosis
Neuman [17] 2014	USA	Retrospective observational studies	56,729 GA 40825 RA 15904	SA/EA	Not mentioned	/
Neuman [5] 2021	USA	Randomized controlled studies	1262 GA 629 RA 633	Single SA	Be determined by the clinical team	Delirium: on the basis of 3D-CAM
Parker [18] 2015	UK	Randomized controlled studies	322 GA 164 RA 158	SA	Be the choice of the anesthetist	Not mentioned
Radcliff [19] 2008	USA	Retrospective observational studies	5683 GA 3353 RA 2330	CEA SA	Not mentioned	Ascertained by the Veterans Health Administration National Surgical Quality Improvement Program data and ICD-9
Seitz [20] 2014	Canada	Retrospective observational studies	12,272 GA 6115 RA 6157	SA	Not mentioned	Not mentioned
Shih [21] 2010	China	Retrospective observational studies	335 GA 167 RA 168	Single SA	Bupivacaine 8-15 mg	Not mentioned
Tung [22] 2016	China	Retrospective observational studies	17,189 GA 11153 RA 6036	SA/EA	Not mentioned	Not mentioned
White [23] 2014	UK	Retrospective observational studies	39,331 GA 15666 RA 23665	SA CSEA SA + NB	Not mentioned	/
White [24] 2016	UK	Retrospective observational studies	2491 GA 985 RA 1506	SA SA + NB	Not mentioned	Deterioration in cognition: abbreviated mental test score
Wood [25] 2011	UK	Retrospective observational studies	1067 GA 489 RA 578	SA	Bupivacaine 0.5% 1.5 ml	/

*GA *General anesthesia, *RA *Regional anesthesia, *NA *Neuraxial anesthesia, *Single SA *Single-injection spinal anesthesia, *SA *Spinal anesthesia, *EA *Epidural anesthesia, *CSA *Continuous spinal anesthesia, *CEA *Continuous epidural anesthesia, *CSEA *Combined spinal epidural anesthesia, *NB *Nerve blocks, *MNB *Multiple nerve blocks, *MI *Myocardial infarction, *ICD-9 *International classification of diseases ninth revision, *ICD-9-CM *International classification of diseases-9-clinical modification, *ACS-NSQIP *The American college of surgeons national surgical quality improvement program, *3D-CAM *3-min diagnostic interview for confusion assessment method, / the study did not included complications

**Table 2:** Complications and findings in included studies

Study (1st author year)	In-hospital mortality		30-day mortality		Pneumonia		Delirium		Findings
	GA	RA	GA	RA	GA	RA	GA	RA	
Basques [7] 2015			450	166	261	109			There was no clear overall advantage of one type of anesthesia over the other
Benjamin [8] 2021	3	5	5	5	2	9			No significant difference
Brox [9] 2016			177	113					No significant difference
Chu [10] 2015	1363	1107							NA was associated with fewer odds of adverse outcomes than GA
Elisabetta [11] 2014	1362	144							Mortality risk did not differ significantly by anesthesia type
Fields [12] 2015					171	65			GA had a higher risk of thirty-day complications as compared to SA
Heidari [13] 2011					0	1			Increased blood loss in GA group
Helwani [14] 2015			20	15					RA was associated with a reduction in deep surgical site infection rates, hospital length of stay, and rates of postoperative cardiovascular and pulmonary complications
Li [4] 2022			4	8	0	1	24	29	RA without sedation did not significantly reduce the incidence of postoperative delirium compared with GA
Linda [15] 2012	9	2							No significant difference
Neuman [16] 2012	325	110			359	153			RA was associated with a lower odds of inpatient mortality and pulmonary complications
Neuman [17] 2014			2197	835					Do not support a mortality benefit for regional anesthesia in this setting
Neuman [5] 2021							124	130	No significant difference
Parker [18] 2015			8	5	3	2	0	3	No significant difference
Radcliff [19] 2008			301	186					GA had a higher risk of 30-day mortality
Seitz [20] 2014			691	665					No significant difference
Shih [21] 2010	5	2			9	3	6	1	GA with an increased risk of postoperative morbidity
Tung [22] 2016			189	104					RA was not associated with 30-day mortality, but was associated with lower 30-day all-cause and surgical site infection readmission compared with GA
White [23] 2014			1066	1713					No significant difference
White [24] 2016			53	87					The type of anesthetic technique is not associated with patient outcome
Wood [25] 2011			23	37					Significant difference in hypotension favoring low dose spinal

*GA *General anesthesia, *RA *Regional anesthesia

### Statistical methods

Review Manager software (Revman for Mac, version 5.3; using the Cochrane Collaboration, Oxford, UK) and Stata statistical software version 12.0 (Stata Corp LP, College Station, TX) were used for meta-analysis. The count data and measurement data were represented by odds ratio (OR) and weighted mean difference (WMD) and their 95% Confidence Interval (CI) representation. If at least two studies reported comparable outcomes, the results of the studies were pooled. This allowed the generation of forest plots, testing for statistical heterogeneity, and the overall estimation of the combined effect of each outcome. The similarity between studies was measured using the I^2^ statistic to estimate the proportion of differences between studies due to heterogeneity rather than chance. For analyzing the heterogeneity, when the between-study heterogeneity was absent in the included studies (I^2^ < 50%), the fixed effect model was used. whereas the random-effects model was applied when between-study heterogeneity was statistically necessary (I^2^ > 50%) [26]. To confirm the reliability of the results in this review, the sensitivity analysis was conducted by the one-by-one elimination method using the Stata. Publication bias was assessed by visual inspection of the funnel plot using the Stata. *P* values < 0.05 were considered statistically significant.

### Assessment of risk of bias

Two investigators (BM and HX) independently assessed the risk of bias in each study. The RCT study applied the randomization research tool of the Cochrane Collaboration [27]. For nonrandomized studies, the Cochrane ACROBAT-NRSI tool [28] was used. Each domain of the Cochrane tool assigned studies as low risk of bias, high risk of bias, or unclear risk of bias.

## Result

### Study selection

**Fig. 1:**
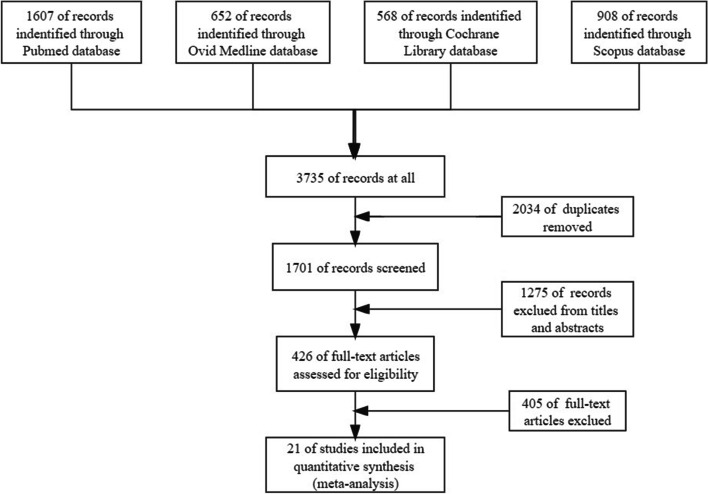
Flow chart presenting the steps of literature search and selection

Firstly, 3735 studies were identified by using PubMed, Ovid Medline, Cochrane Library, and Scopus databases. After removing duplicates, 1701 titles and abstracts were screened. Of these, 1275 were excluded from titles and abstracts. Based on the remaining 426 records, 405 articles were eliminated by reading the full text due to a lack of to be analyzed. Finally, 21 articles were included in the final analysis [4, 5, 7–25], as shown in Fig. 1. The detailed literature search strategies in different databases were displayed in Supplementary material 1.

### Characteristics of eligible studies

The detailed characteristics of the studies were shown in Tables 1 and 2. Among the 21 studies included in the analysis, 17 were retrospective observational studies and 4 were randomized controlled studies [4, 5, 7–25]. Overall, 363,470 patients were analyzed in this systematic review and meta-analysis. 228,713 patients received general anesthesia and 134,757 patients received regional anesthesia. The sample sizes of the included studies varied widely, ranging from 129 to 104,088. Regional anesthesia included the use of a neuraxial technique (spinal anesthesia, epidural anesthesia, continuous spinal anesthesia, continuous epidural anesthesia, or combined spinal-epidural anesthesia) with or without the use of a nerve block or multiple nerve blocks. One study did not provide a definition of regional anesthesia [16].

### Risk of bias

**Fig. 2:**
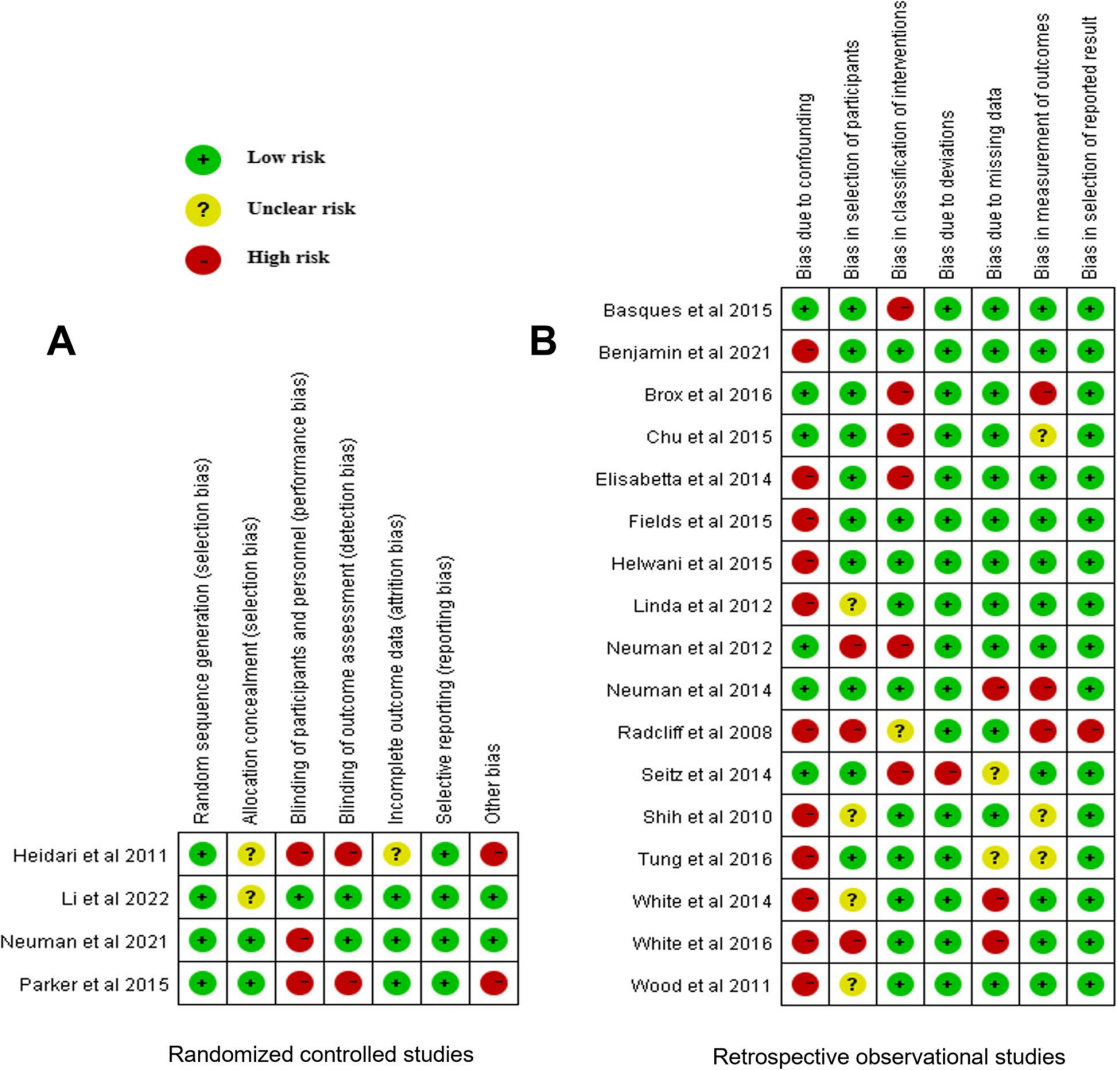
Cochrane collaboration risk of bias for **A** randomized controlled studies and **B** retrospective observational studies

Analysis of the risk of bias for randomized controlled studies and retrospective observational studies are shown in Fig. 2. The authors’ judgments about each risk of bias item for each included study were described. The details were shown in Supplementary material 2 and 3.

### Meta-analysis results

#### In-hospital mortality

**Fig. 3:**
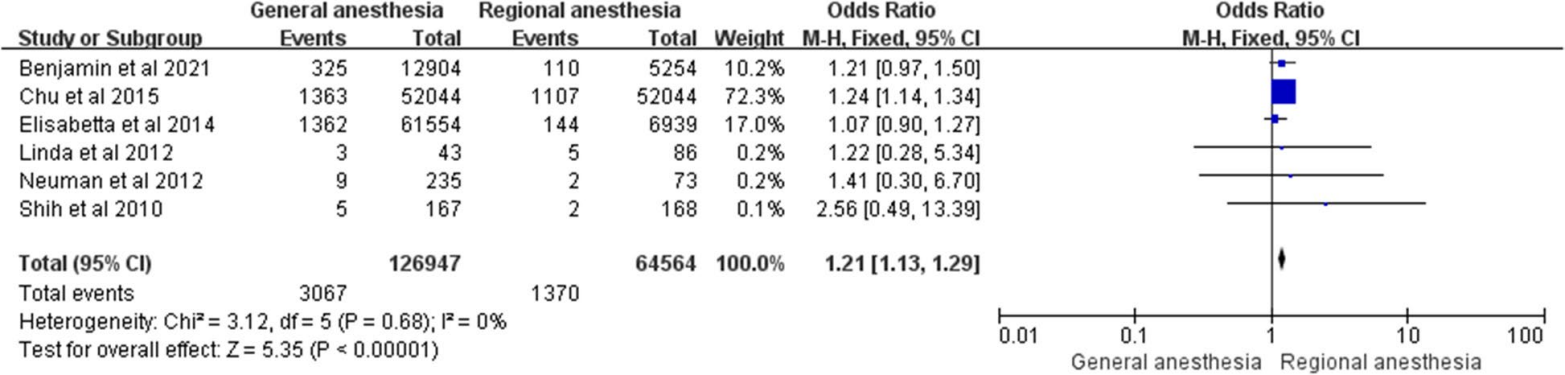
Forest plots showing pooled effect estimates for in-hospital mortality when comparing general with regional anesthesia. The odds ratio was calculated with a fixed effect method

The in-hospital mortality was examined by 6 retrospective observational studies after hip fracture surgery in adults [8, 10, 11, 15, 16, 21]. 4 studies showed that there was no significant difference in in-hospital mortality in patients receiving either general or regional anesthesia [8, 11, 15, 21]. But Neuman and his colleagues (*n* = 18,158, general anesthesia = 12,904, regional anesthesia = 5254) revealed that the in-hospital mortality rate in the general anesthesia group was higher than regional anesthesia group [16]. The study of Chu and his colleagues (*n* = 104,088, general anesthesia = 52,044, regional anesthesia = 52,044) reported a significantly higher incidence of in-hospital mortality in the general anesthesia group [10]. Our meta-analysis, including the above 6 studies, showed a higher in-hospital mortality in the general anesthesia group than in the regional anesthesia group (OR = 1.21; 95% CI 1.13–1.29; *P* < 0.001, *n* = 191,511) without heterogeneity (I^2^ = 0%). The details were shown in Fig. 3.

### 30-day mortality

**Fig. 4:**
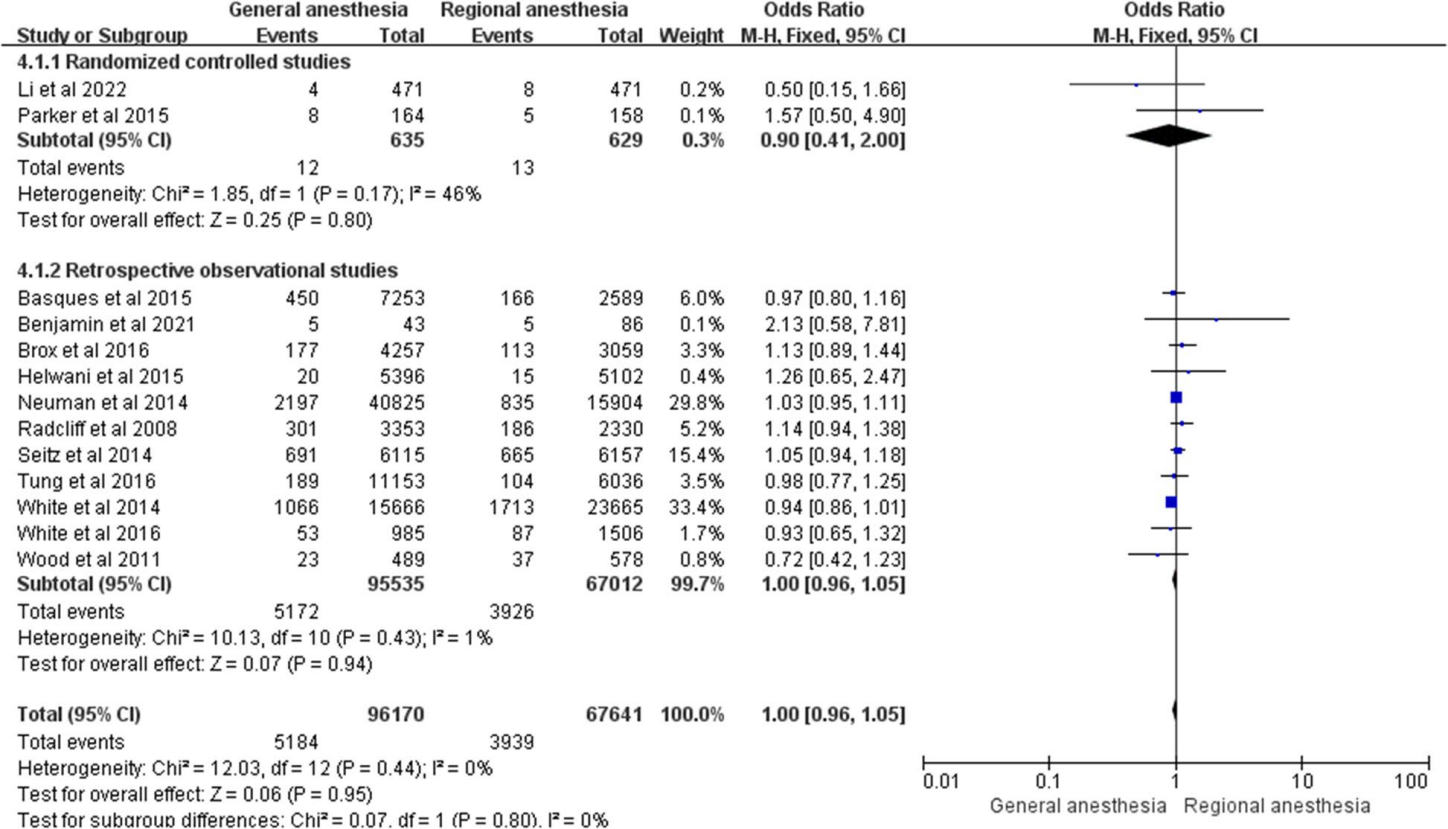
Forest plots showing pooled effect estimates for 30-day mortality when comparing general with regional anesthesia. The odds ratio was calculated with a fixed effect method. 4.1.1: The odds ratio of the randomized controlled studies represented a subgroup. 4.1.2: The odds ratio of the retrospective observational studies represented a subgroup

Thirteen studies investigated the effect of general versus regional anesthesia on 30-day mortality after hip fracture surgery in adult patients including 2 prospective randomized controlled studies and 11 retrospective observational studies [4, 7–9, 14, 17–20, 22–25]. The study of Radcliff and his colleagues (*n* = 5683, general anesthesia = 3353, regional anesthesia = 2330) reported a significantly higher risk of 30-day hospital mortality in the general anesthesia group [19]. The other studies showed that there was no significant difference in the 30-day mortality between general and regional anesthesia in the above studies [4, 7–9, 14, 17, 18, 20, 22–25]. Our meta-analysis of these 13 studies showed no statistically significant difference in 30-day mortality (OR = 1.00; 95% CI 0.96–1.05; *P* = 0.95, *n* = 163,811) without heterogeneity (I^2^ = 0%). The subgroup analysis for 2 randomized controlled studies (OR = 0.90; 95% CI 0.41–2.00; *P* = 0.80, *n* = 1264) and 9 retrospective observational studies (OR = 1.00; 95% CI 0.95–1.04; *P* = 0.89, *n* = 155,797) also indicated no statistical difference in the 30-day mortality between the two groups, as shown in Fig. 4.

### Pneumonia

**Fig. 5:**
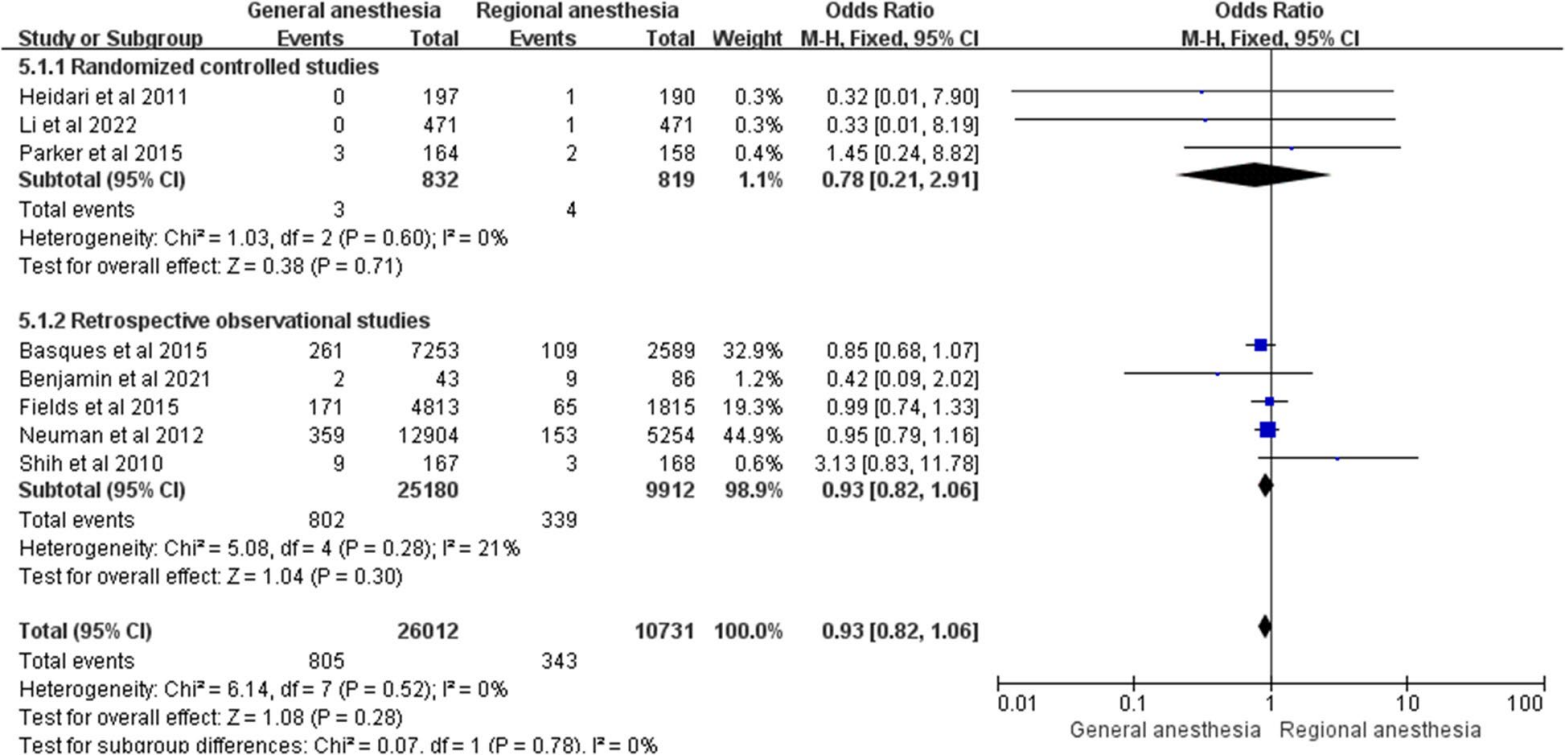
Forest plots showing pooled effect estimates for pneumonia when comparing general with regional anesthesia. The odds ratio was calculated with a fixed effect method. 5.1.1: The odds ratio of the randomized controlled studies represented a subgroup. 5.1.2: The odds ratio of the retrospective observational studies represented a subgroup

Eight studies investigated the incidence of pneumonia after hip fracture surgery in adults, including 3 randomized controlled studies and 5 retrospective observational studies [4, 7, 8, 12, 13, 16, 18, 21]. Among them, Neuman et al. (*n* = 18,158, general anesthesia = 12,904, regional anesthesia = 5254), Fields et al. (*n* = 6628, general anesthesia = 4813, regional anesthesia = 1815) and Shih et al. (*n* = 335, general anesthesia167, regional anesthesia = 168) found a higher incidence of pneumonia in the general anesthesia group [12, 16, 21]. The other studies revealed no significant difference in the incidence of pneumonia between the two groups [4, 7, 8, 13, 18]. These 8 studies were eligible to be included in the meta-analysis. There was no statistical difference in the incidence of pneumonia between the two groups (OR = 0.93; 95% CI 0.82–1.06; *P* = 0.28, *n* = 36,743) without heterogeneity (I^2^ = 0%). The subgroup analysis for 3 randomized controlled studies (OR = 0.78; 95% CI 0.21–2.91; *P* = 0.71, *n* = 1651) and 5 retrospective observational studies (OR = 0.93; 95% CI 0.83–1.06; *P* = 0.30, *n* = 35,092) indicated no statistical difference in the incidence of pneumonia between the two groups, as shown in Fig. 5.

### Delirium

**Fig. 6:**
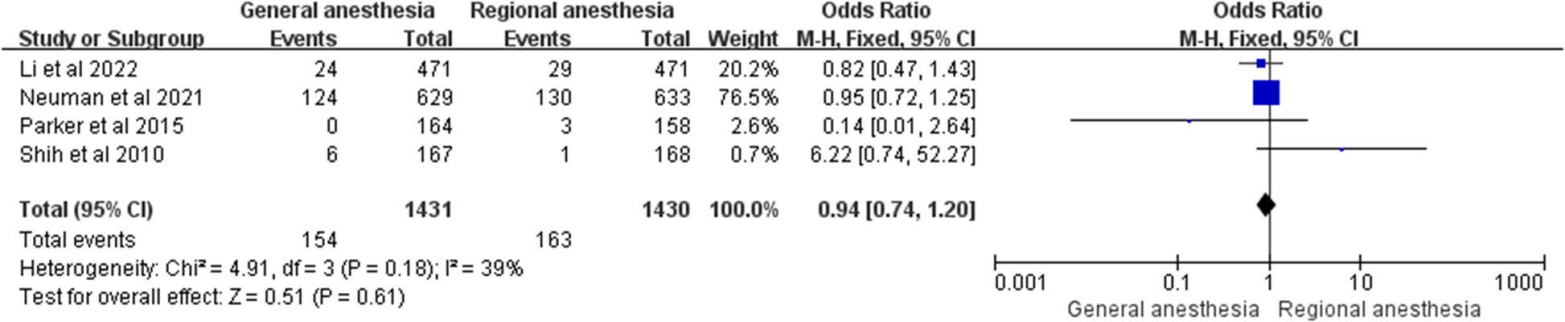
Forest plots showing pooled effect estimates for delirium when comparing general with regional anesthesia. The odds ratio was calculated with a fixed effect method

Three prospective randomized controlled studies and 1 retrospective observational study evaluated the incidence of postoperative delirium between general anesthesia and regional anesthesia in adults. As there was only 1 retrospective study, subgroup analysis was not possible. There was no significant difference in the occurrence of postoperative delirium between general and regional anesthesia [4, 5, 18, 21]. Our meta-analysis of these 4 studies also showed no statistically significant difference between the two groups regarding the incidence of postoperative delirium (OR = 0.94; 95% CI 0.74–1.20; *P* = 0.61, *n* = 2861), without heterogeneity (I^2^ = 39%). The results were shown in Fig. 6.

### Sensitivity analysis

We used Stata 12.0 to perform sensitivity analysis using the one-by-one elimination method. For in-hospital mortality, the combined results of those 6 retrospective observational studies [8, 10, 11, 15, 16, 21] were greatly influenced by the study of Chu et al. [10] (OR = 1.21; 95% CI 1.13–1.29). After excluding this study, the remaining 5 retrospective observational studies [8, 11, 15, 16, 21] indicated there was no significant difference in in-hospital mortality (OR = 1.13; 95% CI 0.99–1.29; *P* = 0.09, *n *= 87,423) by Review Manager software. For 30-day mortality, two prospective randomized controlled studies [4, 18] and 11 retrospective observational studies [7–9, 14, 17, 19, 20, 22–25] showed no significant difference consistently with the original results (OR = 1.00; 95% CI 0.96–1.04). For the occurrence of postoperative pneumonia, 3 randomized controlled studies [4, 13, 18] and 5 retrospective observational studies [7, 8, 12, 16, 21] showed no significant difference between the 2 anesthesia types (OR = 0.93; 95% CI 0.82–1.06). 3 randomized controlled studies and 1 retrospective observational study evaluated the occurrence of postoperative delirium [4, 5, 18, 21] and showed no significant difference (OR = 0.95; 95% CI 0.78–1.16). These results were consistent with the original results indicating that the original results had high reliability (Supplementary material 4).

### Publication bias

**Fig. 7:**
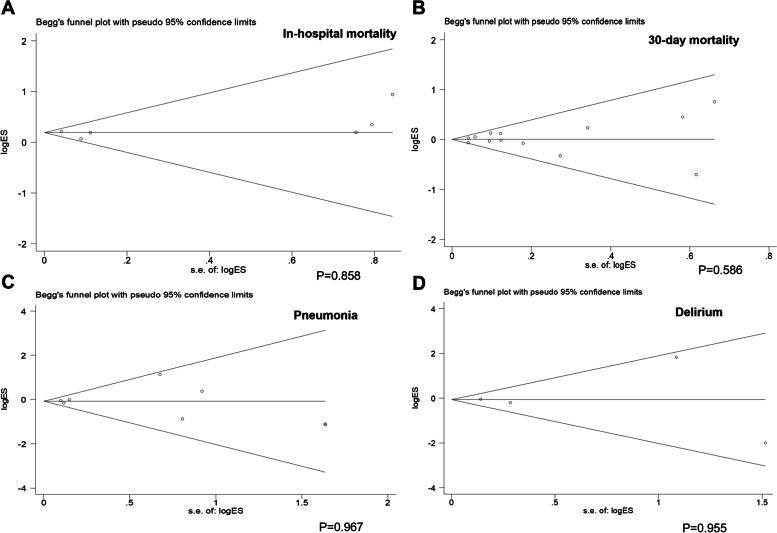
The Begg’s funnel plots of **A** in-hospital mortality, **B** 30-day mortality, **C** pneumonia and **D** delirium suggested that there was no publication bias

Publication bias was assessed by Begg’s test using Stata 12.0. The Begg’s funnel plot of in-hospital mortality(*P* = 0.858), 30-day mortality(*P* = 0.586), the incidence of pneumonia(*P* = 0.967), and delirium(*P* = 0.955) suggested that there was no publication bias, as shown in Fig. 7.

## Discussion

In this systematic review and meta-analysis, we included 21 studies (17 retrospective studies and 4 randomized controlled trials) involving 363,470 patients, of whom 228,713 patients received general anesthesia and 134,757 patients received regional anesthesia. This study showed that general anesthesia was associated with increased in-hospital mortality compared with regional anesthesia in adult patients undergoing hip fracture surgery. There were no significant differences in 30-day mortality, postoperative pneumonia, and delirium in those patients with hip fractures undergoing surgery where either general or regional anesthesia was used. For the outcome of in-hospital mortality, through one-by-one elimination methods, after excluding the study conducted by Chu et al., the outcome has changed to no significant differences in in-hospital mortality. It might be due to the study included 104,088 patients and accounted for 72.3% of the weight affected the outcome. Nevertheless, the result of in-hospital mortality of this meta-analysis was still plausible because there was no heterogeneity and the results showed significant differences for in-hospital mortality (I^2^ = 0, *p* < 0.001). In the future, more large-scale prospective randomized controlled trials might be needed to support the results. Chu et al. found that in hip fracture surgery, the risk factors for in-hospital mortality under general anesthesia compared with regional anesthesia in the elderly may be stroke and acute respiratory failure. Especially patients treated in regional hospitals had greater odds of postoperative stroke after general anesthesia [10]. Neuman et al. and Shin et al. attributed the higher in-hospital mortality under general anesthesia to increased respiratory postoperative complications in older patients [16, 21].

In 2010, Luger and his colleagues conducted a systematic study of the types of anesthesia used in hip fracture surgery. Their systematic review included literature from 1967 to 2010, including 34 randomized studies, 14 observational studies, and 8 systematic reviews and meta-analysis. The authors speculated that spinal anesthesia may be associated with significantly lower early mortality, fewer deep vein thrombotic events, less acute postoperative confusion, less propensity for myocardial infarction, and less pneumonia, fatal pulmonary embolism, and postoperative hypoxia. Because only 18,715 patients were included, the evidence was limited. It might be not suitable to draw definitive conclusions about mortality or other outcomes [29]. In 2016, Guay et al. conducted a systematic review in which they included only 31 randomized controlled studies from 2003 to 2014. There were only 3231 patients were included and only 2152 patients were available for examination of 30-day mortality. They found no difference between the two anesthesia techniques. The authors determined that the number of patients included in the study was insufficient to reveal the differences between general and regional anesthesia in hip fracture patients [30]. In 2017, a systematic review by Van Waesberghe and colleagues had several methodological weaknesses, such as the inclusion of data for patients undergoing elective total hip arthroplasty from ACS-NSQIP databases(America College of Surgeons National Quality Improvement Plan). Many patients from the databases were excluded because of incomplete documentation and many studies did not describe the dose and type of anesthetic. The results showed no difference in 30-day mortality between the two groups of hip fracture surgery patients. The group of nerve block anesthesia could significantly reduce the length of hospital stay and hospital mortality and reduce the incidence of myocardial infarction and respiratory failure [31]. However, In the same year, a systematic review published by O'Donnell and colleagues showed that there were no differences in the 30-day mortality and postoperative complications including the prevalence of pneumonia, acute myocardial infarction, delirium, and renal failure in patients undergoing hip fracture surgery in the two groups [32]. A limitation of the systematic review by O'Donnell and colleagues was that the diagnostic criteria were not standardized and uniform. For example, the definition of delirium was unclear. The patients with a decline in cognitive score in White's study were included in the analysis of delirium [24]. In Ilango's study included, the classification of anesthesia method was unclear and confusing. For example, patients who were sedated under regional anesthesia were recorded as general anesthesia [33]. In our study, we excluded ambiguous groups, such as the group of general anesthesia combined nerve block in Elisabetta's study [11] when in-hospital mortality was analyzed. In terms of 30-day mortality, the groups of general anesthesia combined with epidural or nerve block in the literature were excluded [9, 23, 24]. When analyzing the occurrence of postoperative delirium, we strictly screened the data and excluded the number of cases that did not fully match the definition of delirium, such as cognitive impairment and cognitive score decline, which limited the number of cases collected in the meta.

The systematic review and meta-analysis have several limitations. Firstly, the current evidence lacks high-quality randomized controlled trials, and most of our included studies were retrospective studies. Secondly, perioperative complications are common in elderly hip fracture patients. However, there was a lack of uniform detailed definitions and effective diagnostic criteria for postoperative complications. As a result, most studies were not included in the meta-analysis.

## Conclusions

This review did not show any difference in 30-day mortality and the incidence of postoperative pneumonia and delirium between the general and regional anesthesia groups. The regional anesthesia group was associated with a reduction in in-hospital mortality, but the result was limited by large differences in sample size. More prospective randomized controlled trials are needed in the future. The focus must include clearly defined interventions and outcomes important to patients, as well as unified measurement methods [34] to draw more reliable conclusions.

## Supplementary Information

**Additional file 1.**  Detail literature search strategies in PubMed, OvidMedline, Cochrane Library and Scopusdatabases.

**Additional file 2.** Cochrane collaboration risk of bias for randomized controlled studies.

**Additional file 3.** Cochrane collaboration risk of bias for retrospective observationalstudies.

**Additional file 4.** Sensitivity analyses showing pooled effect estimatesfor (A) In-hospital mortality, (B) 30-day hospital mortality, (C) Pneumonia, (D) Delirium when comparing general anesthesia with regional anesthesia.

## Data Availability

All data relevant to the study are included in the article or uploaded as supplementary information.
