# Supplementary material for: Perioperative outcomes in different anesthesia techniques for patients undergoing hip fracture surgery: a systematic review and meta-analysis
Source: BMC Anesthesiol. 2023 May 27;23:184. doi: 10.1186/s12871-023-02150-9 (PMC10224302; doi:10.1186/s12871-023-02150-9)
Supplement: Supplementary file 1 — Additional file 1. Detail literature search strategies in PubMed, OvidMedline, Cochrane Library and Scopusdatabases. [file 12871_2023_2150_MOESM1_ESM.docx]

Supplementary material 1. Detail literature search strategies in PubMed, Ovid Medline, Cochrane Library and Scopus databases

**PubMed:**

(((anesthesia[Mesh]) OR (anesthesia)) OR (anaesthesia)) AND (((((hip fractures[Mesh]) OR ((hip) AND (fractures))) OR (hip fractures)) OR ((hip) AND (fracture))) OR (hip fracture)) AND ((2002/1/1[PDAT] : 2023/3/31[PDAT]))

**Ovid Medline:**

(((anesthesia[Mesh]) OR (anesthesia)) OR (anaesthesia)) AND (((((hip fractures[Mesh]) OR ((hip) AND (fractures))) OR (hip fractures)) OR ((hip) AND (fracture))) OR (hip fracture)) AND ((2002/1/1[PDAT] : 2023/3/31[PDAT])) full text

**Cochrane Library:**

(Anesthesia or anesthesia or anaesthesia) and (hip fractures or (hip and fracture)) limit to 2002:2023

**Scopus:**

(TITLE-ABS-KEY ( anesthesia OR anesthesia OR anaesthesia ) AND TITLE-ABS-KEY ((hip AND fractures OR ( hip AND fracture)))) AND PUBYEAR > 2003 AND PUBYEAR < 2024 AND (LIMIT-TO ( DOCTYPE, "ar")) AND ( LIMIT-TO (SUBJAREA , "MEDI"))
