# Supplementary material for: Perioperative outcomes in different anesthesia techniques for patients undergoing hip fracture surgery: a systematic review and meta-analysis
Source: BMC Anesthesiol. 2023 May 27;23:184. doi: 10.1186/s12871-023-02150-9 (PMC10224302; doi:10.1186/s12871-023-02150-9)
Supplement: Supplementary file 2 — Additional file 2. Cochrane collaboration risk of bias for randomized controlled studies. [file 12871_2023_2150_MOESM2_ESM.docx]

Supplementary material 2. Cochrane collaboration risk of bias for randomized controlled studies.


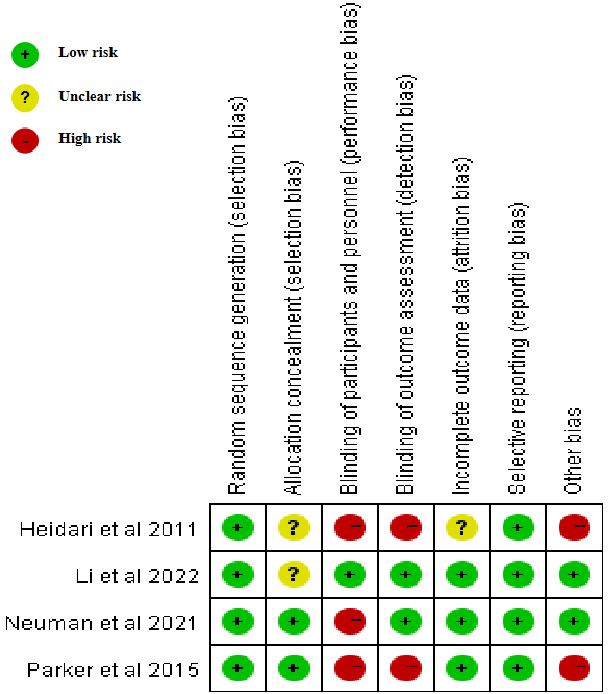


**Heidari et al 2011**

| Risk of bias | Author’s judgement | Support for judgement |
| --- | --- | --- |
| Random sequence generation (selection bias) | Low risk | Randomization process explained |
| Allocation concealment (selection bias) | Unclear risk | No information |
| Blinding of participants and personnel (performance bias) | High risk | No information |
| Blinding of outcome assessment (detection bias) | High risk | No information |
| Incomplete outcome data (attrition bias) | Unclear risk | All missing data accounted for |
| Selective reporting (reporting bias) | Low risk | All results presented as per method |
| Other bias | High risk | Not based on intention-to-treat principle: patients excluded because of change in anaesthetic/ surgical plan |

**Li et al 2022**

| Risk of bias | Author’s judgement | Support for judgement |
| --- | --- | --- |
| Random sequence generation (selection bias) | Low risk | Randomization process explained |
| Allocation concealment (selection bias) | Unclear risk | No information |
| Blinding of participants and personnel (performance bias) | Low risk | Data collectors were blinded to group allocation throughout the study. |
| Blinding of outcome assessment (detection bias) | Low risk | Trained outcome assessors were blinded |
| Incomplete outcome data (attrition bias) | Low risk | All missing data accounted for |
| Selective reporting (reporting bias) | Low risk | All results presented as per method |
| Other bias | Low risk | Groups well balanced |

**Neuman 2021**

| Risk of bias | Author’s judgement | Support for judgement |
| --- | --- | --- |
| Random sequence generation (selection bias) | Low risk | Randomization process explained |
| Allocation concealment (selection bias) | Low risk | Permuted block randomization  with variable block sizes. |
| Blinding of participants and personnel (performance bias) | How risk | No blinding of patient or participant |
| Blinding of outcome assessment (detection bias) | Low risk | Outcome were obtained by trial staff who were unaware of the treatment assignments |
| Incomplete outcome data (attrition bias) | Low risk | All missing data accounted for |
| Selective reporting (reporting bias) | Low risk | All results presented as per method with no deviation from intended intervention |
| Other bias | Low risk | Groups well balanced |

**Parker 2015**

| Risk of bias | Author’s judgement | Support for judgement |
| --- | --- | --- |
| Random sequence generation (selection bias) | Low risk | Randomization process explained |
| Allocation concealment (selection bias) | Low risk | Sealed envelope |
| Blinding of participants and personnel (performance bias) | High Risk | No blinding of patient or participant |
| Blinding of outcome assessment (detection bias) | High risk | No outcome assessor blinding |
| Incomplete outcome data (attrition bias) | Low risk | All data accounted for; only 1 patient lost to follow up |
| Selective reporting (reporting bias) | Low risk | All outcomes reported as per method and conversion to GA or spinal within the intervention groups stated clearly with intention to treat principle |
| Other bias | High risk | More male patients in GA group |
