# Supplementary material for: Perioperative outcomes in different anesthesia techniques for patients undergoing hip fracture surgery: a systematic review and meta-analysis
Source: BMC Anesthesiol. 2023 May 27;23:184. doi: 10.1186/s12871-023-02150-9 (PMC10224302; doi:10.1186/s12871-023-02150-9)
Supplement: Supplementary file 3 — Additional file 3. Cochrane collaboration risk of bias for retrospective observationalstudies. [file 12871_2023_2150_MOESM3_ESM.docx]

Supplementary material 3. Cochrane collaboration risk of bias for retrospective observational studies.


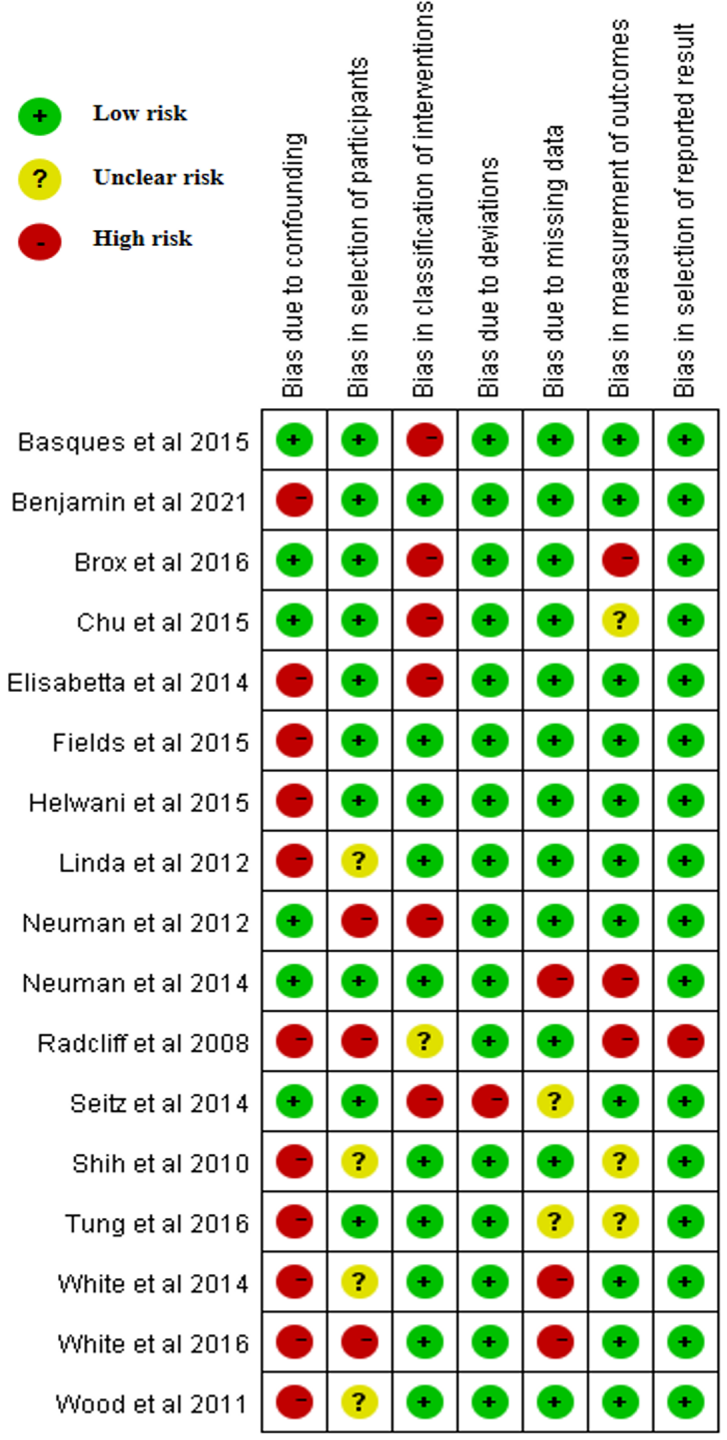


**Basques et al 2015**

| Bias | Author’s judgment | Support for judgement |
| --- | --- | --- |
| Bias due to confounding | Low risk | The authors used calculated propensity scores to mitigate the selection. |
| Bias in the selection of participants | Low risk | Clear inclusion/exclusion criteria |
| Bias in the classification of interventions | High risk | The ACS-NSQIP database does not capture the type or anaesthetic dosage used |
| Bias due to deviations from intended intervention | Low risk | There were no departures from intended interventions |
| Bias due to missing data | Low risk | No important data missing |
| Bias in measurement of outcomes | Low risk | The database was filled with data from medical records and interviews by trained reviewers |
| Bias in selection of reported result | Low risk | The authors used bivariate and propensity-adjusted multivariate regression analyses. Binary outcomes were compared using logistic regression |

**Benjaminet al 2021**

| Bias | Author’s judgment | Support for judgement |
| --- | --- | --- |
| Bias due to confounding | High risk | Inherent risk with observational studies |
| Bias in the selection of participants | Low risk | Clear inclusion/exclusion criteria |
| Bias in the classification of interventions | Low risk | Interventions defined |
| Bias due to deviations from intended intervention | Low risk | No cross-over of interventions, three distinct groups |
| Bias due to missing data | Low risk | All data accounted for |
| Bias in measurement of outcomes | Low risk | Standardised methods of outcome measurement for the groups |
| Bias in selection of reported result | Low risk | All results reported as per method |

**Brox 2016**

| Bias | Author’s judgment | Support for judgement |
| --- | --- | --- |
| Bias due to confounding | Low risk | The authors used Pearson’s chisquared test and the Kruskal-Wallis test to mitigate selection bias |
| Bias in the selection of participants | Low risk | Clear inclusion/exclusion criteria |
| Bias in the classification of interventions | High risk | The database does not capture the type or dose of anaesthetic used |
| Bias due to deviations from intended intervention | Low risk | There were no departures from intended interventions |
| Bias due to missing data | Low risk | No important data missing |
| Bias in measurement of outcomes | High risk | No information about the people collecting the data |
| Bias in selection of reported result | Low risk | The authors used a multivariable conditional logistic regression model |

**Chu et al 2015**

| Bias | Author’s judgment | Support for judgement |
| --- | --- | --- |
| Bias due to confounding | Low risk | The author used calculated propensity score to mitigate the selection bias |
| Bias in the selection of participants | Low risk | Clear inclusion/exclusion criteria |
| Bias in the classification of interventions | High risk | The database does not capture the type or dose of anaesthetic used. |
| Bias due to deviations from intended intervention | Low risk | There were no departures from intended interventions |
| Bias due to missing data | Low risk | All data accounted for |
| Bias in measurement of outcomes | Unclear risk | A database was used without information of the people |
| Bias in selection of reported result | Low risk | The author used a propensity score, Student t test, Pearson chi-square test |

**Elisabetta et al 2014**

| Bias | Author’s judgment | Support for judgement |
| --- | --- | --- |
| Bias due to confounding | High risk | No attempt to address potential confounding factors |
| Bias in the selection of participants | Low risk | Clear inclusion/exclusion criteria |
| Bias in the classification of interventions | High risk | Interventions poorly defined |
| Bias due to deviations from intended intervention | Low risk | Groups analysed according to intervention with no cross-over |
| Bias due to missing data | Low risk | All data accounted for |
| Bias in measurement of outcomes | Low risk | Methods of outcome assessment same for each group |
| Bias in selection of reported result | Low risk | All results reported as per method |

**Fields et al 2015**

| Bias | Author’s judgment | Support for judgement |
| --- | --- | --- |
| Bias due to confounding | High risk | Propensity score matching to address potential confounding factors |
| Bias in the selection of participants | Low risk | Clear inclusion/exclusion criteria |
| Bias in the classification of interventions | Low risk | Interventions defined |
| Bias due to deviations from intended intervention | Low risk | Groups analysed according to intervention with no cross-over |
| Bias due to missing data | Low risk | All data accounted for |
| Bias in measurement of outcomes | Low risk | Outcomes standardly reported in the same way for both groups |
| Bias in selection of reported result | Low risk | All results reported as per method |

**Helwani et al 2015**

| Bias | Author’s judgment | Support for judgement |
| --- | --- | --- |
| Bias due to confounding | High risk | Retrospective study with high risk of confounders |
| Bias in the selection of participants | Low risk | Clear inclusion/exclusion criteria |
| Bias in the classification of interventions | Low risk | Interventions defined |
| Bias due to deviations from intended intervention | Low risk | There were no departures from intended interventions |
| Bias due to missing data | Low risk | No important data missing |
| Bias in measurement of outcomes | Low risk | Dedicated data personnel collect, validate and submit the data after rigorous uniform training  and examination |
| Bias in selection of reported result | Low risk | Demographic and clinical characteristics were compared between the two groups by using Pearson chi-square test for all categorical variables |

**Linda et al 2012**

| Bias | Author’s judgment | Support for judgement |
| --- | --- | --- |
| Bias due to confounding | High risk | Retrospective study with high risk of confounders |
| Bias in the selection of participants | Unclear risk | Exclusion criteria not clearly defined |
| Bias in the classification of interventions | Low risk | Interventions well defined |
| Bias due to deviations from intended intervention | Low risk | Distinct groups analysed according to intervention with no crossover |
| Bias due to missing data | Low risk | No important data missing |
| Bias in measurement of outcomes | Low risk | Outcomes reported in the same way for each group |
| Bias in selection of reported result | Low risk | All results reported as per method |

**Neuman et al 2012**

| Bias | Author’s judgment | Support for judgement |
| --- | --- | --- |
| Bias due to confounding | Low risk | The author used calculated propensity scores to mitigate selection bias |
| Bias in the selection of participants | High risk | The two groups were retrospectively determined according to anaesthesia technique |
| Bias in the classification of interventions | High risk | The study does not describe the dose and type of anaesthetic used |
| Bias due to deviations from intended intervention | Low risk | No departure from intervention |
| Bias due to missing data | Low risk | No missing data |
| Bias in measurement of outcomes | Low risk | The results were overseen by the U.S. Agency for Healthcare |
| Bias in selection of reported result | Low risk | The author used the Wilcoxon rank sum test and the chi-square test to compare the results |

**Neuman et al 2014**

| Bias | Author’s judgment | Support for judgement |
| --- | --- | --- |
| Bias due to confounding | Low risk | The authors used nearfar matching, standardized differences, across-hospitalmatch and a within-hospital match to reduce the selection bias |
| Bias in the selection of participants | Low risk | Clear inclusion/exclusion criteria |
| Bias in the classification of interventions | Low risk | Interventions defined |
| Bias due to deviations from intended intervention | Low risk | No departure from intervention |
| Bias due to missing data | High risk | Data excluded in the matching process unaccounted for |
| Bias in measurement of outcomes | High risk | No information about the way the results were collected |
| Bias in selection of reported result | Low risk | All results reported as per method |

**Radcliff et al 2008**

| Bias | Author’s judgment | Support for judgement |
| --- | --- | --- |
| Bias due to confounding | High risk | Retrospective study with high risk of confounders |
| Bias in the selection of participants | High risk | The two groups were retrospectively determined according to anaesthesia technique |
| Bias in the classification of interventions | Unclear risk | Interventions poorly defined |
| Bias due to deviations from intended intervention | Low risk | No departure from intervention |
| Bias due to missing data | Low risk | All data accounted for |
| Bias in measurement of outcomes | High risk | Unclear how the data were collected and how the clinical measurement was done |
| Bias in selection of reported result | High risk | Not all results reported |

**Seitz et al 2014**

| Bias | Author’s judgment | Support for judgement |
| --- | --- | --- |
| Bias due to confounding | Low risk | The authors used calculated propensity scores to mitigate the selection bias |
| Bias in the selection of participants | Low risk | Clear inclusion/exclusion criteria |
| Bias in the classification of interventions | High risk | The study does not describe the dose and type of anaesthetic used |
| Bias due to deviations from intended intervention | High risk | Every case that was not purely a spinal was placed in the general anaesthesia group |
| Bias due to missing data | Unclear risk | Missing data not mentioned |
| Bias in measurement of outcomes | Low risk | The used data sets were linked using unique, encoded identifiers |
| Bias in selection of reported result | Low risk | The authors used the Wilcoxon rank-sum test and chi-square test to compare the results |

**Shih et al 2010**

| Bias | Author’s judgment | Support for judgement |
| --- | --- | --- |
| Bias due to confounding | High risk | No attempt to address potential confounding factors |
| Bias in the selection of participants | Unclear risk | Inclusion criteria not clearly defined |
| Bias in the classification of interventions | Low risk | Interventions defined |
| Bias due to deviations from intended intervention | Low risk | Patients who were not exclusively in one group were excluded |
| Bias due to missing data | Low risk | All data accounted for |
| Bias in measurement of outcomes | Unclear risk | Unclear how information on outcomes was collected |
| Bias in selection of reported result | Low risk | All results reported as per method |

**Tung et al 2016**

| Bias | Author’s judgment | Support for judgement |
| --- | --- | --- |
| Bias due to confounding | High risk | No attempt to address potential confounding factors |
| Bias in the selection of participants | Low risk | Clear inclusion/exclusion criteria |
| Bias in the classification of interventions | Low risk | Interventions defined |
| Bias due to deviations from intended intervention | Low risk | No deviation from intended intervention |
| Bias due to missing data | Unclear risk | No mention of missing data |
| Bias in measurement of outcomes | Unclear risk | Unclear how causes for readmission were ascertained |
| Bias in selection of reported result | Low risk | All major readmission specific causes listed as per methods |

**White et al 2014**

| Bias | Author’s judgment | Support for judgement |
| --- | --- | --- |
| Bias due to confounding | High risk | Retrospective study with high risk of confounders |
| Bias in the selection of participants | Unclear risk | Unclear inclusion/exclusion criteria |
| Bias in the classification of interventions | Low risk | Interventions defined |
| Bias due to deviations from intended intervention | Low risk | No departure from intervention |
| Bias due to missing data | High risk | Missing data not accounted for |
| Bias in measurement of outcomes | Low risk | Data were collected by specially trained personnel employed by each eligible hospital |
| Bias in selection of reported result | Low risk | The authors used a twotailed chi-squared test without Yate’s correction and multivariable regression analysis |

**White et al 2016**

| Bias | Author’s judgment | Support for judgement |
| --- | --- | --- |
| Bias due to confounding | High risk | Retrospective study with high risk of confounders |
| Bias in the selection of participants | High risk | The two groups were retrospectively determined according to anaesthesia technique |
| Bias in the classification of interventions | Low risk | Interventions defined |
| Bias due to deviations from intended intervention | Low risk | No departure from intervention |
| Bias due to missing data | High risk | 16904 patient records. However only 11085 could be analysed |
| Bias in measurement of outcomes | Low risk | Data were collected by specially trained personnel employed by each eligible hospital |
| Bias in selection of reported result | Low risk | The authors used Fisher’s exact test, chi-squared, Wilcoxon and Haenzel tests |

**Wood et al 2011**

| Bias | Author’s judgment | Support for judgement |
| --- | --- | --- |
| Bias due to confounding | High risk | No attempt to account for confounding factors |
| Bias in the selection of participants | Unclear risk | Exclusion criteria not mentioned |
| Bias in the classification of interventions | Low risk | Interventions well defined |
| Bias due to deviations from intended intervention | Low risk | No departure from intervention |
| Bias due to missing data | Low risk | No missing data |
| Bias in measurement of outcomes | Low risk | Outcomes reported in the same way for each group |
| Bias in selection of reported result | Low risk | All results reported as per method |
