# Supplementary material for: Perioperative outcomes in different anesthesia techniques for patients undergoing hip fracture surgery: a systematic review and meta-analysis
Source: BMC Anesthesiol. 2023 May 27;23:184. doi: 10.1186/s12871-023-02150-9 (PMC10224302; doi:10.1186/s12871-023-02150-9)

Supplementary material 4. Sensitivity analyses showing pooled effect estimates for (A) In-hospital mortality, (B) 30-day hospital mortality, (C) Pneumonia, (D) Delirium when comparing general anesthesia with regional anesthesia.

**A In-hospital mortality**


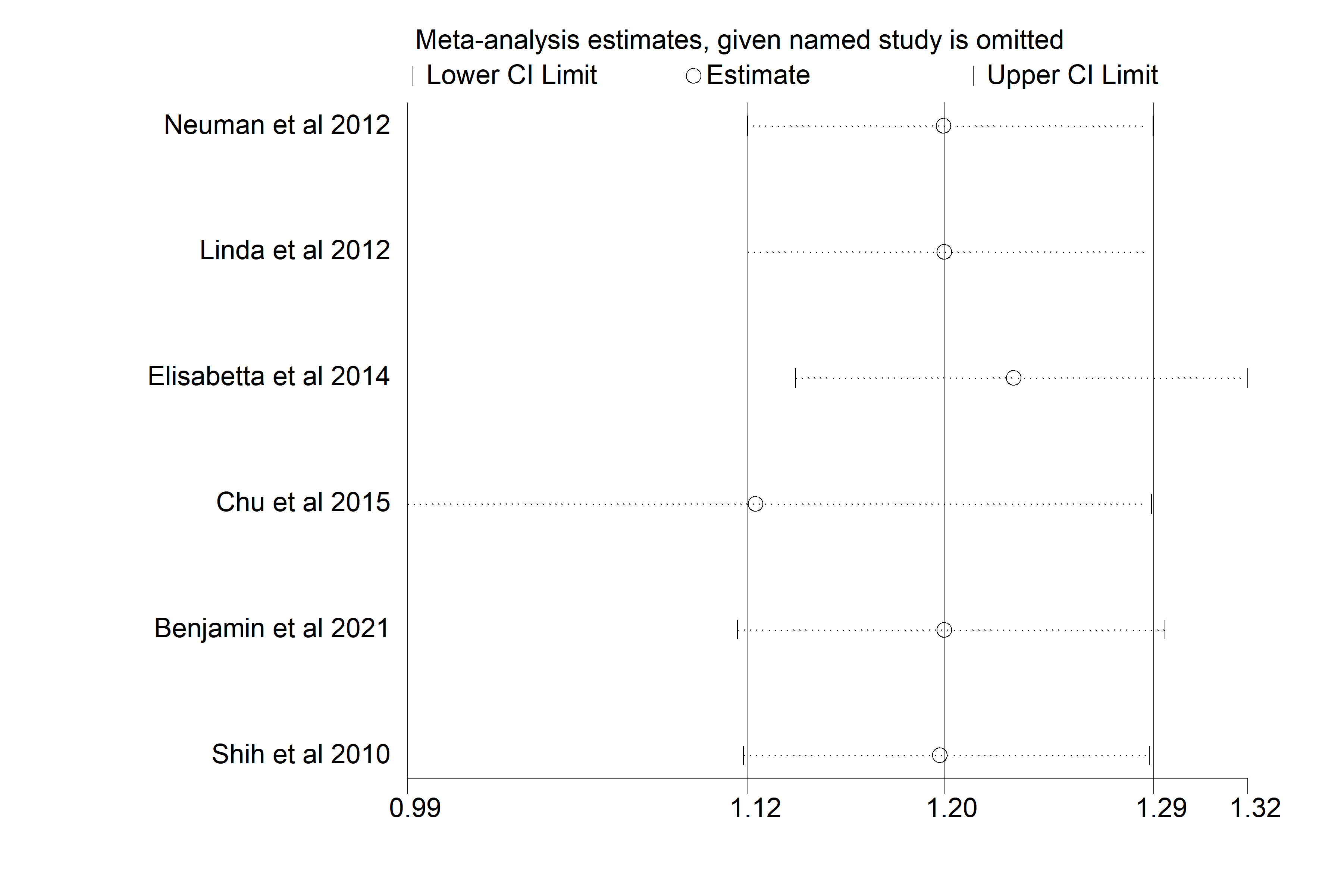


**B 30-day hospital mortality**


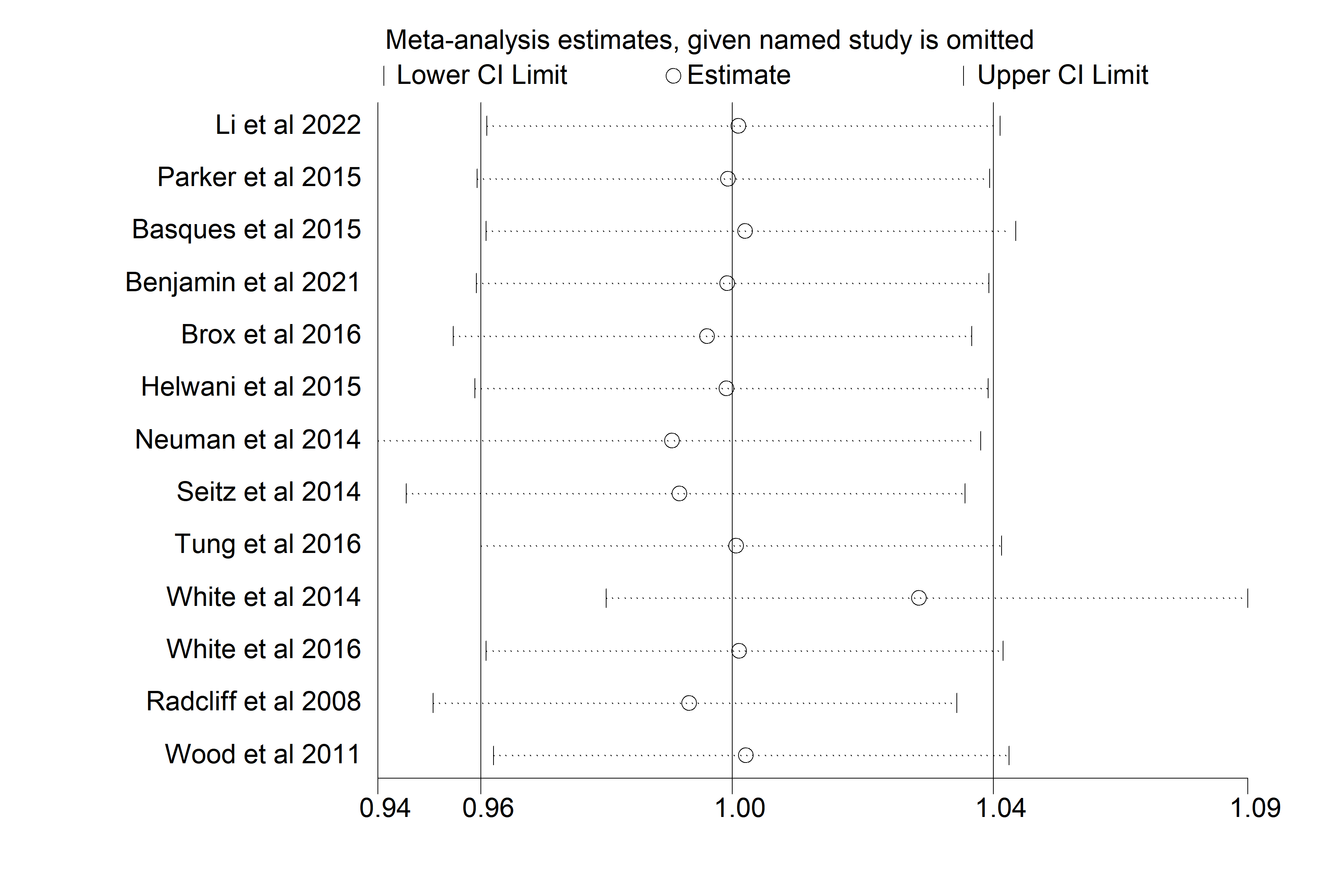


**C Pneumonia**


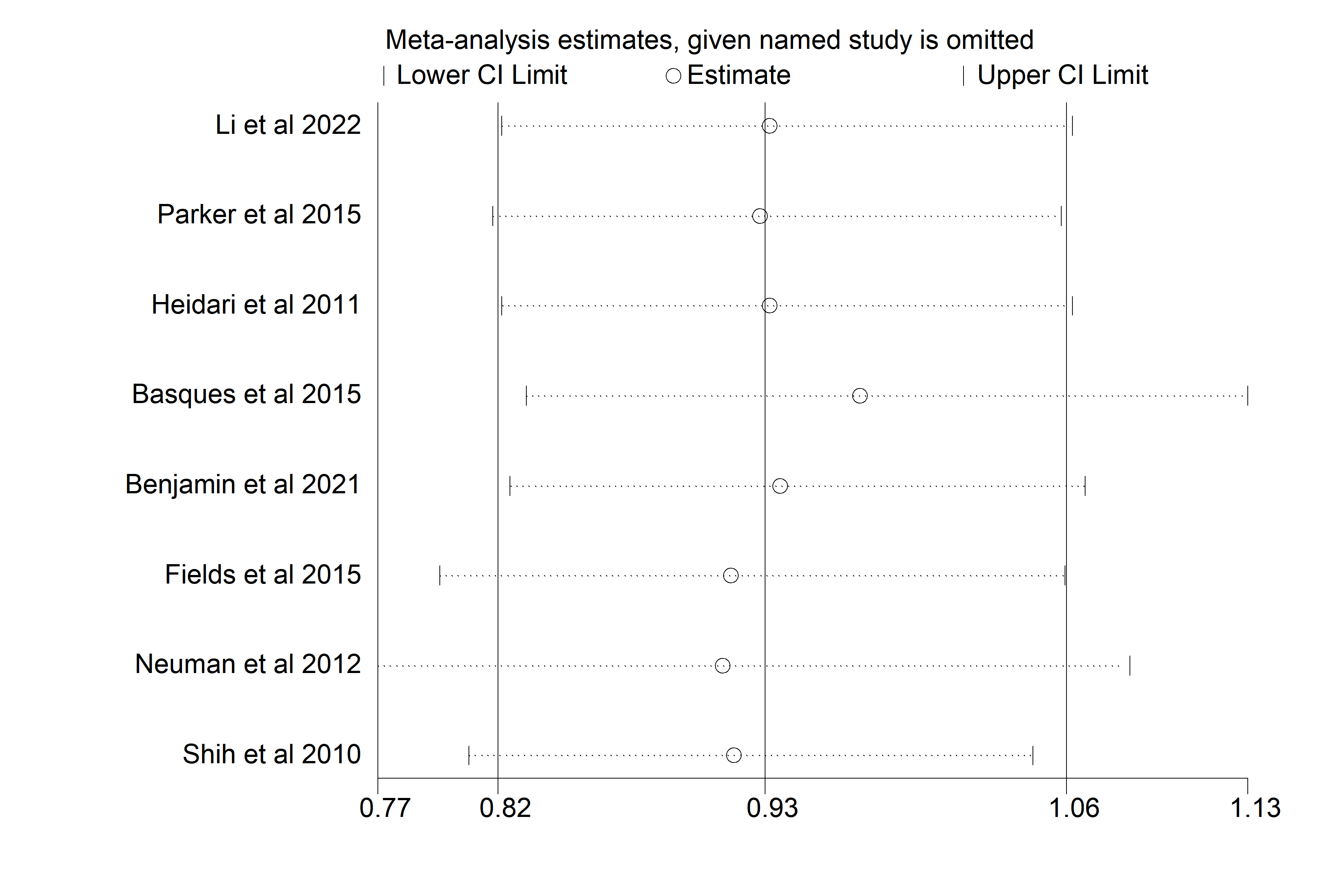


**D Delirium**


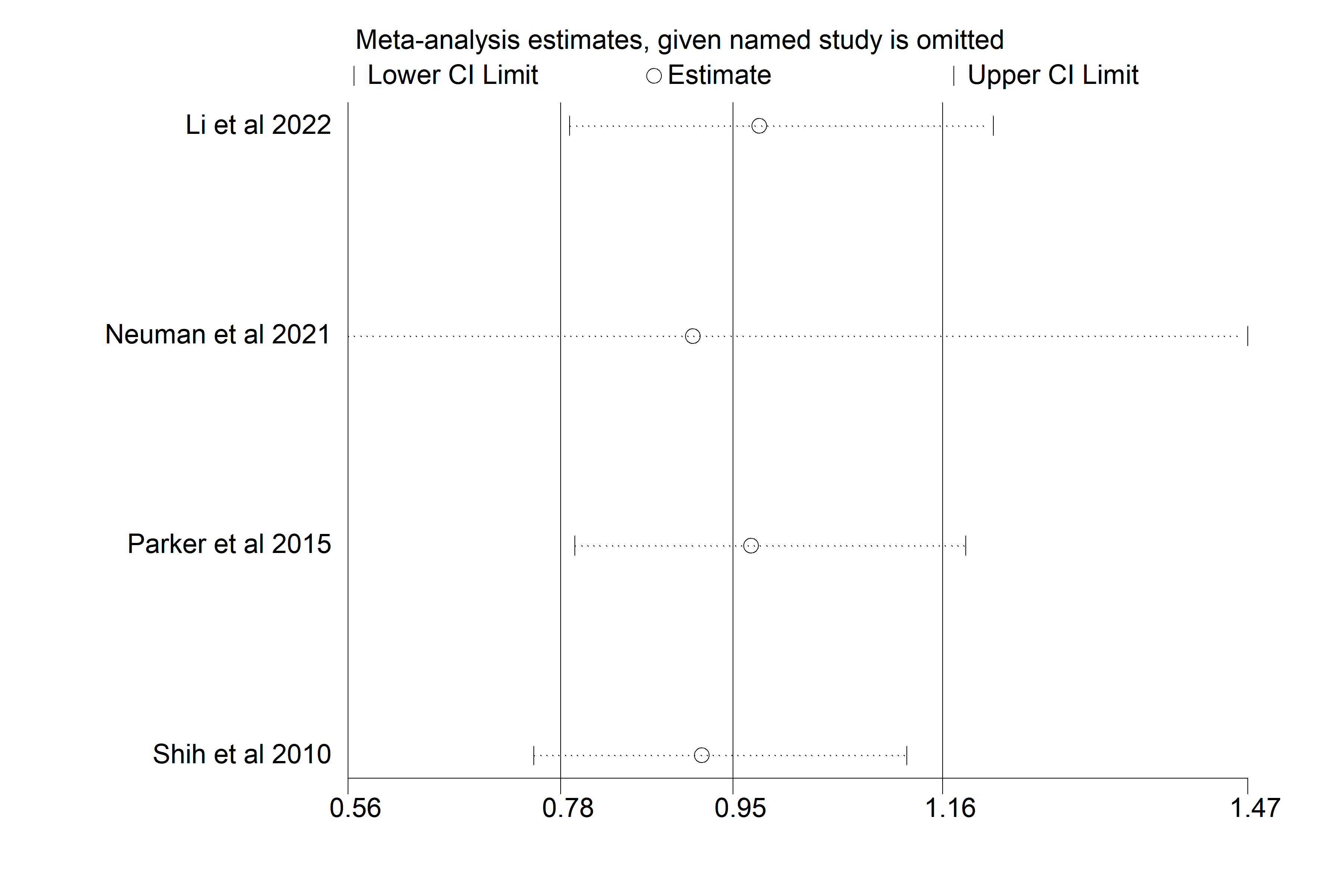

Supplement: Supplementary file 4 — Additional file 4. Sensitivity analyses showing pooled effect estimatesfor (A) In-hospital mortality, (B) 30-day hospital mortality, (C) Pneumonia, (D) Delirium when comparing general anesthesia with regional anesthesia. [file 12871_2023_2150_MOESM4_ESM.doc]
